# TREC-IN: gene knock-in genetic tool for genomes cloned in yeast

**DOI:** 10.1186/1471-2164-15-1180

**Published:** 2014-12-24

**Authors:** Suchismita Chandran, Vladimir N Noskov, Thomas H Segall-Shapiro, Li Ma, Caitlin Whiteis, Carole Lartigue, Joerg Jores, Sanjay Vashee, Ray-Yuan Chuang

**Affiliations:** The J. Craig Venter Institute, 9704 Medical Center Drive, Rockville, 20850 MD USA; INRA, UMR 1332 de Biologie du Fruit et Pathologie, F-33140 Villenave d’Ornon Bordeaux, France; UMR 1332 de Biologie du Fruit et Pathologie, University Bordeaux, F-33140 Villenave d’Ornon Bordeaux, France; International Livestock Research Institute (ILRI), Old Naivasha Road, PO Box 30709, 00100 Nairobi, Kenya

**Keywords:** Mycoplasma, TREC, Gene knock-in, Genomes, Yeast, Autonomously replicating sequence, Genome minimization

## Abstract

**Background:**

With the development of several new technologies using synthetic biology, it is possible to engineer genetically intractable organisms including *Mycoplasma mycoides* subspecies *capri* (*Mmc*), by cloning the intact bacterial genome in yeast, using the host yeast’s genetic tools to modify the cloned genome, and subsequently transplanting the modified genome into a recipient cell to obtain mutant cells encoded by the modified genome. The recently described tandem repeat coupled with endonuclease cleavage (TREC) method has been successfully used to generate seamless deletions and point mutations in the mycoplasma genome using the yeast DNA repair machinery. But, attempts to knock-in genes in some cases have encountered a high background of transformation due to maintenance of unwanted circularization of the transforming DNA, which contains possible autonomously replicating sequence (ARS) activity. To overcome this issue, we incorporated a split marker system into the TREC method, enabling seamless gene knock-in with high efficiency. The modified method is called TREC-assisted gene knock-in (TREC-IN). Since a gene to be knocked-in is delivered by a truncated non-functional marker, the background caused by an incomplete integration is essentially eliminated.

**Results:**

In this paper, we demonstrate applications of the TREC-IN method in gene complementation and genome minimization studies in *Mmc*. In the first example, the *Mmc dnaA* gene was seamlessly replaced by an orthologous gene, which shares a high degree of identity at the nucleotide level with the original *Mmc* gene, with high efficiency and low background. In the minimization example, we replaced an essential gene back into the genome that was present in the middle of a cluster of non-essential genes, while deleting the non-essential gene cluster, again with low backgrounds of transformation and high efficiency.

**Conclusion:**

Although we have demonstrated the feasibility of TREC-IN in gene complementation and genome minimization studies in *Mmc*, the applicability of TREC-IN ranges widely. This method proves to be a valuable genetic tool that can be extended for genomic engineering in other genetically intractable organisms, where it may be implemented in elucidating specific metabolic pathways and in rationale vaccine design.

**Electronic supplementary material:**

The online version of this article (doi:10.1186/1471-2164-15-1180) contains supplementary material, which is available to authorized users.

## Background

Mycoplasmas are the simplest and smallest living prokaryotes (0.1 μm), and although phylogenetically related to Gram-positive bacteria, lack a cell wall [1]. They also have the smallest recorded genomes (0.58 Megabases (Mb) – 1.38 Mb) for bacterial species that can replicate autonomously, and have colonized a wide range of hosts including, humans and animals [2]. However, efforts to manipulate mycoplasma genomes are fraught with difficulties owing to the lack of genetic tools available for these organisms [3]. This has made understanding the biology and elucidating the host-pathogen mechanism for any potential therapeutics, including vaccine development, challenging.

One of the early genetic tools that were developed for understanding mycoplasma biology was the generation of *OriC* plasmids that could replicate in mycoplasma cells [4–6]. Although heterologous gene expression and targeted gene disruption by single-crossover recombination were demonstrated in *Mycoplasma mycoides* subspecies *capri* (*Mmc*) and *M. capricolum* subspecies *capricolum* (*Mcc*), no recombination events were observed in the closely related *M. mycoides* subspecies *mycoides* (*Mmm*) [7, 8]. In addition, maintaining stable mutants using *OriC* plasmids turned out to be difficult and laborious [4, 7, 8]. Thus, alternate strategies were designed, including a transposon-based method to generate mutants with low passage numbers that were free of antibiotic-resistance genes [9]. Transposon-based mutagenesis has been prevalently used as a genetic tool in mycoplasmas to generate mutants of interest as well as to define essential genes required for survival [10–12]. Furthermore, a double-crossover homologous recombination method using a suicide plasmid has been described for *M. genitalium*, albeit at a very low frequency [13–15], but this method did not address seamless deletion and removal of markers [16]. Therefore, to overcome stability and marker recycling issues, we turned to yeast genetics and synthetic biology to extend the genetic toolbox of mycoplasmas. With recent advancements in synthetic genomics including, cloning of the *Mmc* genome in yeast, manipulation of the mycoplasma genome using yeast genetic tools, transplantation of the engineered mycoplasma genome from yeast to a bacterial recipient cell, and creation of the synthetic cell, expression of the engineered genome became possible [17–22]. Mycoplasma genomes including *M. genitalium* (0.6 Mb), *M. pneumonia*e (0.8 Mb), and *Mmc* (1.1 Mb) were first cloned into yeast with the idea of implementing yeast genetics tools to engineer genetically intractable organisms [17–22].

**Figure 1:**
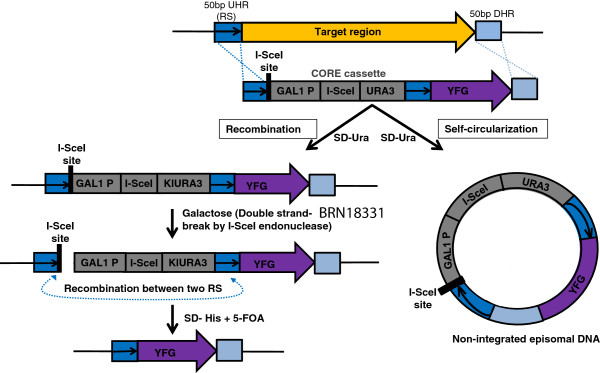
**Brief outline of TREC and background formation.** The gene to be inserted (your favorite gene, YFG), indicated by a purple arrow can be seamlessly inserted into a target site (orange arrow) via TREC. The knock-in sequence (purple arrow) is placed immediately downstream of the repeat sequence (RS) (dark blue box containing a black arrow) in the CORE cassette (gray boxes). After integration, induction of double strand break can promote homologous recombination between the two repeat sequences, leading to removal of the CORE cassette, as shown on the left. However, a fraction of transforming DNA may circularize itself through illegitimate recombination or non-homologous end joining, where broken DNA ends join. The resulting DNA may be maintained as a non-integrated plasmid if the knock-in sequence contains ARS activity, as shown on the right. UHR, indicates upstream homology region, and DHR, downstream homology region.

Once cloned in yeast, bacterial genomes can be theoretically manipulated by yeast genetic tools. The URA3 marker/5-FOA counter-selection is a common technique in which the marker can be recycled to create seamless gene deletions, replacements, or gene knock-ins. However, we have previously shown that this conventional two-step method was very inefficient in engineering a mycoplasma genome cloned in yeast due to instability of the genome where high background of 5-FOA resistant colonies resulted from non-specific removal of the URA3 marker [22]. Development of the tandem repeat coupled with endonuclease cleavage (TREC) method has greatly improved the efficiency of seamless gene deletions [21, 22]. TREC can be also applied in gene knock-in via a single step transformation where the knock-in sequence is placed outside the cassette and immediately next to the repeated sequence (Figure 1). The removal of the cassette leaves the knock-in sequence in the target site seamlessly. Although TREC method is currently the best tool that can seamlessly engineer a genome cloned in yeast [21, 22], the process is sometimes inefficient with a high background of transformation, arising possibly due to illegitimate recombination (Figure 1). To overcome this limitation of TREC-mediated gene insertion, we developed a modified method, called TREC assisted gene knock-in (TREC-IN) that significantly improves the efficiency of gene knock-in and vastly reduces screening effort. This method relies on the split marker system whereby the gene is delivered by a non-functional truncated antibiotic resistant gene module *kanMX*, and a site-specific gene insertion is selected by functional restoration of the full length *kanMX* gene. Here, we demonstrate the feasibility of TREC-IN in the *Mmc* genome using two examples a) replacement of an endogenous gene with an orthologous one, and b) essential gene complementation in a genome reduction study.

## Results

### Design of the TREC-IN

**Figure 2:**
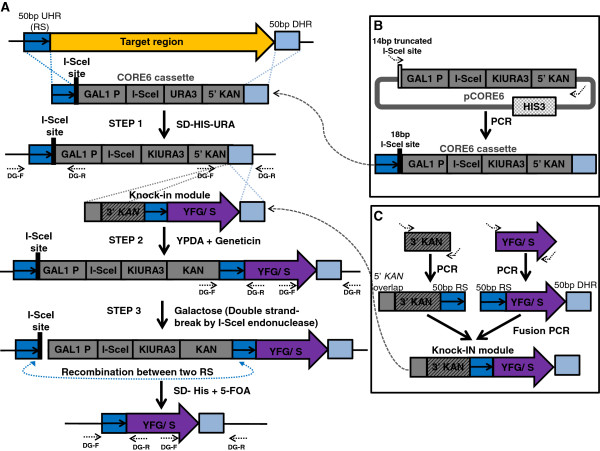
**Schematic representation of TREC-IN. (A)** Outline of the TREC-IN method. Step 1: The target region (orange arrow) is replaced with a CORE6 cassette by homologous recombination via two 50-bp homology sequences [upstream homology region (UHR) or repeat sequence (RS), indicated by dark-blue box containing a black arrow, and downstream homology region (DHR), indicated by light-blue box]. Transformed cells were selected for Uracil prototrophy. The cassette includes the reconstituted I-SceI binding site (black bar), and the I-Sce1 gene under the control of the Gal1 promoter, a Uracil marker, and 5’ *kanMX* gene componenet. Step 2: The knock-in module [containing your favorite gene/ sequence (YFG/S, purple arrow) and the 3’ region of the *kanMX* gene component, interspersed by the 50 bp RS, and flanked by 50 bp homology region to the 5’ *kanMX* gene component in the cassette and DHR to the target site] is transformed and selected on geneticin resistance for full complementation of the *kanMX* gene component. Step 3: Yeast colonies are grown in presence of galactose to express I-SceI, which produces a double-strand break at the I-SceI site, and enhances homologous recombination (dashed double-headed arrow) between the two RS, resulting in excision of the CORE6 and knock-in modules. Colonies are grown on 5-FOA for Uracil counter selection. Primers to confirm correct insertions are shown by dashed arrows, and synthetic primers are represented by kinked arrows. **(B)** CORE6 cassette construction. The CORE6 cassette is amplified from pCORE6 to add four nucleotides (tagg) for full reconstitution of the I-SceI binding site (black bar). 50 bp UHR and 50 bp DHR, specific to the target site, are also included in the construction. **(C)** Knock-in module construction. The knock-in module is constructed by a PCR-based fusion method. The two amplicons are the 3’ *kanMX* gene component carrying the 3’ region of kanamycin gene (gray striped box), the RS, and a homology region to the 5’ region of the kanamycin gene in CORE6 (gray box), and the replacement gene (YFG/S) flanked by the RS and DHR.

The design of TREC-IN is based on the previous TREC strategy and incorporates a split marker approach with an additional step for a gene knock-in that is mediated by a functional restoration of the kanamycin resistance gene module*, kanMX*. The procedure involves three steps: first, insertion of a CORE6 cassette to the target locus; second, site-specific gene integration; and third, seamless cassette recycling (Figure 2A). In the first step, the CORE6 cassette, which consists of the 18 bp I-SceI binding site, the I-SceI endonuclease encoding gene under the control of the yeast *GAL1* promoter, the *KlURA3* gene and a 5′ truncated *kanMX* gene component, is introduced to the target site. Similar to the TREC method, two sequences of about 50 bp that are homologous to the target site are added into the CORE6 cassette by PCR on 5′ and 3′ ends of the cassette so that they flank the CORE6 cassette (Figure 2B). Transformation of the CORE6 cassette into yeast and homologous recombination at the target site results in the replacement of the target site by the CORE6 cassette. Transformed yeast colonies are selected for uracil prototrophs, and further analyzed by PCR screening to confirm that the homologous recombination has occurred at the correct target site (Figure 2A). The second step of TREC-IN involves construction and transformation of the knock-in module containing a 3′ truncated *kanMX* gene component and the knock-in sequence. The kanamycin resistance gene and the knock-in sequence are separated by a repeat sequence of about 50 bp in length, which is identical to upstream sequences of the target site in the CORE6 cassette (Figure 2C). This knock-in module is flanked at the 5′ end by a region of the kanamycin resistance gene to allow for homologous recombination at the 3′ end of the CORE6 cassette. On its 3′ end, the knock-in module is flanked by the same homologous region that is also present on the 3′ end of the CORE6 cassette to allow for recombination at the target site (Figure 2A). Upon transformation, the knock-in module integrates into the target site, resulting in an insertion containing two repeat sequences encompassing three genes (the I-SceI*, KlURA3*, and the full length *kanMX* module) and the knock-in sequence. Transformed yeast colonies are selected for resistance against the antibiotic geneticin, and then analyzed by PCR screening to confirm correct insertion. In the third step of TREC-IN, the whole cassette flanked by the two repeat sequences is removed via homologous recombination between the two repeat sequences. The efficiency of the recombination is enhanced by the double strand break (DSB) generated by the cleavage of the endonuclease I-SceI at the 18 bp recognition site in the cassette after galactose induction. The removal of the *KlURA3,* I-SceI*,* and the *kanMX* module counter-selected by 5-FOA would leave no scar. Only the knock-in sequence remains at the target site. Yeast cells that are resistant to 5-FOA are screened by PCR for the precise insertion of the replacement sequence (Figure 2).

### Replacement of the *Mcc*orthologous *dnaA*gene in the *Mmc*genome

**Figure 3:**
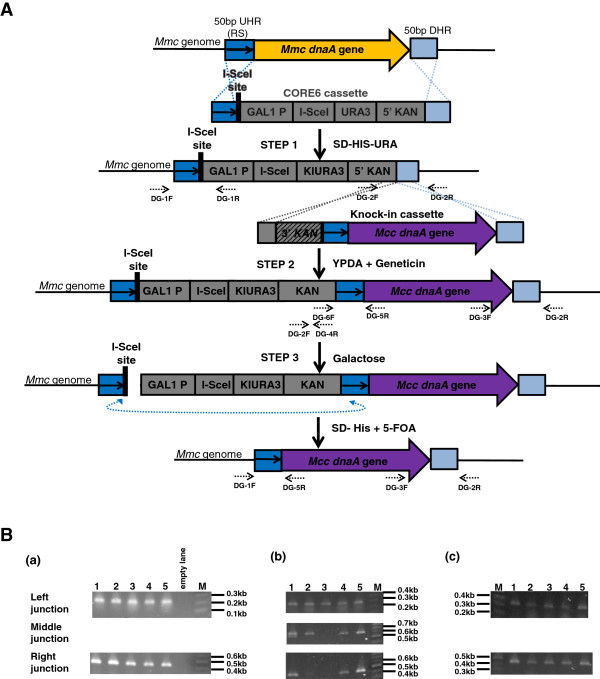
**Replacement of** ***Mcc dnaA*** **gene in the mycoplasma genome. (A)** Schematic representation of replacement of the *Mmc dnaA* gene with an orthologous gene from *Mcc*. Here, the *dnaA* gene in the *Mmc* genome (indicated by an orange arrow) was replaced by the *Mcc* orthologue (purple arrow) using TREC-IN. Diagnostic primers to confirm correct insertion of the cassettes and seamless replacement of the endogenous *dnaA* gene are indicated by dashed arrows (see Additional file 1: Figure S3 for primer information). **(B)** PCR screening to confirm replacement of the *Mmc dnaA* gene with an orthologous gene from *Mcc*. (a) DNA from yeast colonies after selection on SD-His-Ura were amplified using primers DG1F/DG1R (left junction; expected size, 222 bp) and DG2F/DG2R (right junction; expected size, 438 bp). (b) DNA from yeast colonies after selection on geneticin were amplified using primers DG2F/DG4R (left junction; expected size, 285 bp), DG6F/DG5R (middle junction; expected size, 615 bp) and DG3F/DG2R (right junction; expected size, 446 bp). (c) DNA from yeast colonies after selection on 5-FOA were amplified using primers DG1F/DG5R (left junction; expected size, 388 bp) and DG3F/DG2R (right junction; expected size, 446 bp). A representative of 5 colonies is shown for each transformation and for each junction.

To demonstrate precise replacement of an orthologous gene in the *Mmc* genome, TREC-IN was applied to replace the *Mmc dnaA* gene, which is essential for chromosomal replication and viability [5, 19], with the orthologous *dnaA* gene from *Mcc*. The *Mmc* (accession no. AY277700) and *Mcc dnaA* genes (accession no. D90426) share 95% sequence identity at both the nucleotide and protein levels (analyzed using BLAST). As described in Methods, the first step of TREC-IN resulted in the precise replacement of the endogenous *dnaA* gene by the CORE6 cassette (Figure 3A, step 1). The promoter and 3′ region of the *Mmc dnaA* gene were left unaltered. In the second step of TREC-IN, the orthologous *Mcc dnaA* gene was integrated downstream of the CORE6 cassette under geneticin selection (Figure 3A, step 2). In the third step, DSB at the I-SceI site promoted homologous recombination between the two repeat sequences, followed by precise and seamless insertion of the *Mcc dnaA* gene in the *Mmc* genome (Figure 3A, step 3). Each step of the deletion and replacement procedure was evaluated by PCR screening to confirm the correct insertions and junctions (Figure 3B).

Upon transformation, seven colonies were screened by PCR and all the colonies were found to be positive for CORE6 replacement [Figure 3B (a)]. In the second step, we PCR-screened 36 geneticin-resistant colonies for multiple junctions and found that 33/36 (>91%) of the colonies were positive for the precise insertion of the cassette at the targeted locus [Figure 3B (b)]. In the third step, I-SceI-mediated DSB resulted in 27/36 (75%) of the colonies showing precise removal of the cassette, resulting in a clean insertion [Figure 3B (c)]. Of note, PCR-screening [Figure 3B (b) and 3B (c)] indicated that while majority of the colonies obtained resulted in seamless replacement of the knock-in gene, the remaining 9% and 25% colonies respectively, were positive for only one or two of the junctions tested, suggesting non-specific recombination. Thus, TREC-IN proves to be a valuable genetic tool to overcome background issues and facilitate gene knock-in experiments with increased efficiency.

Both, the *dnaA* gene-deleted and *dnaA* gene-complemented *Mmc* genomes were transplanted to generate the mutant *Mmc* strains, as described previously [21]. As expected, genome transplantation of the *dnaA* gene-deleted genome resulted in non-viability. However, replacement of the orthologous *Mcc dnaA* gene resulted in a viable cell. The resulting colonies were of similar size to those of the control wild-type *Mmc* colonies (data not shown). Genomic DNA from the *dnaA-*replaced *Mmc* cells was isolated and analyzed by sequencing to confirm the precise and scar-less insertion of the *Mcc dnaA* gene.

### Application of TREC-IN for genome reduction in the *Mmc*genome

**Figure 4:**
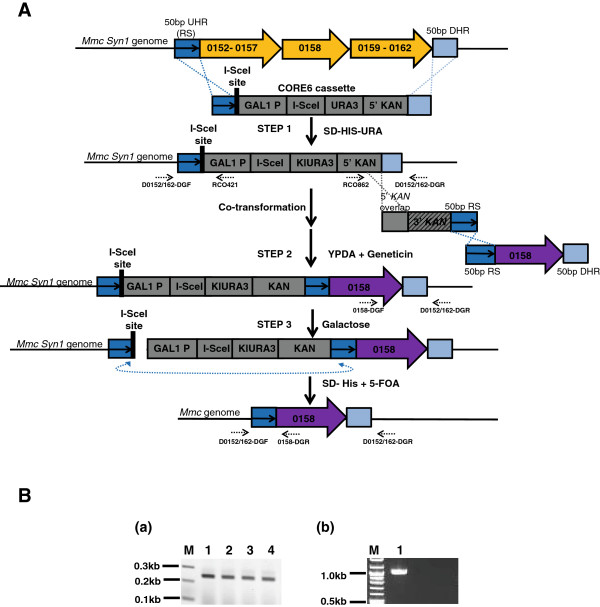
**Cluster deletion and replacement of an essential gene using TREC-IN. (A)** Schematic of replacement of the intervening *ssrA* essential gene (0158) upon cluster deletion in *Mmc* Syn1. In step 1, the target region (orange arrows) containing the *ssrA* gene and two adjacent non-essential clusters (0152-0157 and -159-0162) were replaced with the CORE6 cassette. In step 2, a knock-in module was integrated into the target site by co-transforming two PCR products. One of the amplicons contained the *ssrA* gene, 50 bp RS, and 50 bp DHR. The other amplicon included the 3’ region of the *kanMX* split marker gene component, 50 bp RS, and 50 bp homology region to the 5’ *kanMX* gene component in the CORE6 cassette. Homologous recombination resulted in full complementation of the *kanMX* gene component. Yeast colonies were selected for geneticin resistance and then grown on galactose. In step 3, galactose induces I-SceI expression, which produces double-strand break at the I-SceI site and enhances intra-molecular homologous recombination (dashed double-headed blue arrow) between the two RS, resulting in excision of the CORE6 cassette. Colonies were grown on 5-FOA for Uracil counter selection. **(B)** PCR screening to confirm cluster deletion and replacement of the *ssrA* essential gene. **(a)** DNA from four yeast colonies after selection on 5-FOA were amplified using diagnostic primers (dashed arrows) D0152/162-DGF and 0158-DGR (left junction; expected size, 239bp), and **(b)** D0152/162-DGF and D0152/162-DGR (expected size of *ssrA* replacement, 1.0kb).

Global transposon random mutagenesis has been widely used to identify non-essential genes in minimal genome studies in prokaryotes [10–12]. Therefore, using transposons, we generated a high-resolution map of non-essential gene candidates on a synthetic *Mmc* genome (unpublished data). To carry out a top-down genome reduction strategy, consecutive non-essential genes were grouped into multigene deletion targets and were labeled non-essential gene clusters (NEGC). In some cases, several NEGCs were interspersed by single or a few Tn5-defined essential genes. To achieve genome reduction more efficiently, the TREC-IN approach was tested to remove multiple NEGCs simultaneously, and then add back the essential genes to the genome that were interspersed between them. To demonstrate this application, we chose a 16 kb region of the synthetic *Mmc* genome (*Mmc* Syn1) covering two NEGCs consisting of 10 genes, separated by a Tn5-denfined essential gene (*ssrA*) for deletion (Figure 4A and Additional file 1: Figure S2). In the first step of TREC-IN, the integration of the CORE6 cassette at the target site resulted in the deletion of the two NEGCs (*Mmc* Syn1 0152-0157 and *Mmc* Syn1 0159-0162) along with the intervening essential gene *ssrA* (*Mmc* Syn1 0158) from the *Mmc* Syn1 genome. In the second step of TREC-IN, transformation of the knock-in module resulted in the precise insertion of the *Mmc ssrA* gene back into the synthetic *Mmc* genome. The precise cluster deletion followed by insertion of the *ssrA* gene was verified by PCR screening (Figure 4B). The phenotypes of both cluster-deleted and *ssrA* gene-complemented *Mmc* strains were determined by genome transplantation. We found that the whole 16 kb deletion comprising 11 genes resulted in a non-functional genome as observed by the lack of viable cells. However, cis-complementation of the *ssrA* gene rescued the lethal phenotype. Transplantation colonies from the *ssrA* complemented synthetic *Mmc* genome were viable, and showed similar colony size to those of the control synthetic *Mmc* cells (data not shown). Genomic DNA from the *ssrA* complemented *Mmc* cells was isolated and analyzed. Sequencing of the complemented *ssrA* region in the isolated modified synthetic *Mmc* genome confirmed the precise and seamless insertion of the essential *Mmc ssrA* gene and deletion of the two NEGCs.

## Discussion

Mycoplasmas infect a wide range of hosts, including humans and animals, and in some cases, even contribute towards economic havoc [1, 2]. Therefore, developing better genetic tools to study and contain these pathogens has become a priority. *Mmc*, with its relatively small genome, and ease of manipulation [20, 21] is not only being probed as a model to study pathogenesis, but also as a model organism to test the concept of a minimal cell, where essential genes and functions are being determined. Additionally, *Mmc* is also being modeled as a platform to develop tools towards vaccine development that can be applied to other mycoplasma species. However, the existing genetic tool box makes it difficult to study this bacterial species.

With recent advancements in synthetic genomics, it is now possible to engineer the *Mmc* genome using yeast genetic tools, including TREC [17–22]. Development of TREC is based on a modified yeast system where generation of seamless deletions [22] and point mutations (unpublished data) in the mycoplasma genome is now made possible by using the yeast DNA repair machinery. In principle, the TREC method can be employed to insert genes of interest into the *Mmc* genome. However, several attempts to knock-in an *Mmc* gene into the *Mmc* genome were inefficient with a high background of transformation. Since yeast ARSs are A-T rich, and the mycoplasma genome is relatively A-T rich, it is reasonable to speculate that gene knock-ins containing A-T rich sequences of mycoplasma genomes likely contain ARS activity [17]. Akada and colleagues reported that a gene containing an ARS performs inefficient chromosomal integration [23]. Thus, a portion of the transforming DNA can circularize through illegitimate recombination or NHEJ [24], and be maintained as a non-integrated free plasmid in the yeast cell (unpublished results, CL). To circumvent this problem, we developed TREC-IN, which can efficiently produce gene knock-ins without leaving any scars. Since the TREC-IN method encompasses elements of the TREC method and a split marker system for seamless replacement of nucleotide sequences at any given location on the genome, background issues arising from unwanted ARS activity and A-T rich content are greatly reduced. In the example of *dnaA* gene replacement (Figure 2) TREC-IN was employed to replace the A-T rich *Mmc* gene with an orthologous gene from *Mcc.* However in this case, direct comparison with TREC was not possible because the orthologous genes share a high degree of homology (95%) and the TREC design would not be able to resolve partial recombination occurring between the two genes, as expected. Therefore by using TREC-IN, efficiency of replacement is vastly improved with frequencies of obtaining a positive clone nearing 75% (see Results), thus circumventing the cumbersome screening process of TREC which would be labor and time intensive. Of note, comparing efficiencies between TREC and TREC-IN proves to be complicated as it varies on a case-case basis where A-T content and secondary structure has to be taken into consideration.

Furthermore, TREC-IN can also been extended to delete genes with possible ARS-like activity from the *Mmc* genome, which are very difficult to achieve by the TREC method. For example, we made several attempts to delete the glycerol facilitator (*glpF*) gene from the *Mmc* genome using TREC (unpublished results). We found transformations yielded increased levels of background colonies growing on selective media, without the correct replacement. Yet, when the TREC-IN strategy was applied to delete the *Mmc glpF* gene, all colonies obtained contained the precise and seamless deletion of the *glpF* gene (manuscript in preparation, SC, LM, CL, JJ, RC, SV). In contrast to the TREC method, no background colonies were observed. While TREC-IN depends upon integration of the CORE cassette to the target site, there is some flexibility in choosing the integration sites. Depending on the case, the design can be modified to target sequences that may reside in upstream or downstream adjacent genes if the original target sites prove to be difficult. The adjacent genes can then be restored by including the deleted sequence in the knock-in fragment. The *glpF* deletion described above provides such an example. In this case, the design for the downstream homologous region (Figure 2) was modified to include part of the neighboring *glpK* gene, in order to bypass a specificity issue at the 3′ end of the *glpF* gene. The missing *Mmc glpK* region was complemented in the second step of the TREC-IN method.

TREC has been employed to delete target sites of greater than 70 kb seamlessly in *Mmc* (unpublished results), and since TREC-IN utilizes elements of the TREC, it can be speculated that TREC-IN can also be used to seamlessly delete similarly large nucleotide tracts. Although analysis of knock-in sequences larger than 3 kb has not been carried out, it is theoretically possible that TREC-IN would be able to handle larger fragments, possibly with lower efficiency in trying to complete homologous recombination. In summary, TREC-IN proved to be useful in modifying regions of the genome that tend to be difficult to engineer either due to high A-T content or ARS activity, where TREC or other conventional yeast genetic tools maybe limiting.

## Conclusion

The TREC-IN method proves to be a powerful genetic tool for manipulating mycoplasma genome. In addition to finding applications in our top-down genome minimization of *Mmc* (Figure 4), this method can be employed to explore homologous complementation studies in other related organisms, including *M. leachii*, and *M. putrefaciens* efficiently without the cumbersome screening process that would be required by TREC alone. By using TREC-IN to manipulate metabolic pathways, pathogenic and virulence factors may be studied with relative ease; thereby facilitating better vaccine design against some of the economically devastating livestock diseases such as contagious bovine pleuropneumonia caused by mycoplasmas [25]. In our studies of mycoplasma biology, TREC and TREC-IN dramatically increased our ability to manipulate the genomes of genetically intractable bacteria. As synthetic genomics techniques are extended to other bacteria that are difficult to manipulate genetically, TREC and TREC-IN will become even more valuable as tools for engineering bacterial genomes cloned as yeast centromeric plasmids.

## Methods

### Yeast strain and media

The yeast *Saccharomyces cerevisiae, strain* VL6-48 (*MATαhis3-Δ200 trp1-Δ1 KlURA3-Δ1 lys2 ade2-101 met14*) containing either the 1.08 Mb genome of *Mycoplasma mycoides* subspecies *capri* (*Mmc*) with a yeast centromeric plasmid (YCP) [21], or the synthetic *Mmc* genome (*Mmc* Syn1) [19] were employed. Yeast cells were grown and maintained in either the synthetic minimal medium containing dextrose (SD) [21], or the standard rich medium containing glucose (YPD) or galactose (YPG) [22]. SD medium was supplemented with 5-fluoroorotic acid (5-FOA), for KlURA3 counter-selection [21, 26].

### Preparation of mutagenesis cassettes

#### A. Construction of pCORE6 plasmid

The pCORE6 plasmid (GenBank accession number KP282615) was constructed by cloning the 5′ region of the kanamycin resistance gene along with its promoter (5′ *KanMX,* 1-859 bp) into the previously constructed pCORE3 plasmid (unpublished) at the EcoR I site (Additional file 1: Figure S1). More precisely, 5′ *kanMX* was amplified from the previously described pFA6a-KanMX plasmid [27] using primers, RCO858 (CAG*GAATTC*GACATGGAGGCCCAGAATAC) and RCO859 (ATC*GAATTC*GGCCAGCCATTACGCTCGT), containing the EcoR I restriction site (*GAATTC*) at each extremity. The pCORE3 plasmid, which includes a 14 bp incomplete I-SceI binding site (white box), a Gal1 promoter, an I-SceI gene, and yeast *KlURA3* prototrophic gene (gray boxes), was linearized with EcoR I. This plasmid also contains a *HIS3* gene and can be selected for histidine autotrophy (Additional file 1: Figure S1).

The pCORE3 plasmid and the 5′ *kanMX* amplified product were then ligated to form pCORE6 (Additional file 1: Figures S1 and S4). In these constructions, the I-SceI restriction site is maintained in a truncated form (**GATAACAGGGTAAT**) (white bar) because leaky expression of the I-SceI endonuclease (if the plasmid is propagated in *Escherichia coli)*, would result in cleavage of the pCORE3 and pCORE6 plasmids at the I-SceI site. Therefore, four additional nucleotide sequences (tagg) must be added to restore the complete 18 bp I-SceI site during amplification of the CORE6 knock-out cassette (black bar) (Figure 2A,B) (see below).

#### B. Preparation of mutagenesis cassette for the replacement of *Mmc dnaA*gene by *Mcc dnaA*gene in *Mmc*genome

A modified version of the CORE cassette described previously [22] was constructed as follows. Briefly, the CORE6 cassette includes an 18 bp I-SceI binding site (black bar), followed by a *GAL1* promoter, I-SceI endonuclease gene, and *KlURA3* gene (gray boxes). The CORE6 contains an additional sequence, which includes the 5′ region of the kanamycin resistance gene component (5′ *kanMX*) [Promoter for the Translation Elongation Factor (PTEF) followed by the 5′ kanamycin resistance gene sequence (1 to 859 bp)], which forms part of the split marker system (Figure 2, Additional file 1: Figures S1 and S4). The CORE6 cassette was amplified by polymerase chain reaction (PCR) from the plasmid pCORE6 (Figure 2B) using the chimeric primers, CORE6-F (*GTT TTC CAC ATT TTT AAC AAGTGT TTA ACT ATA ATATTT TTG GAG ACA AAT* tagg**GATAA CAG GGT AAT**ACG GAT TA) and CORE6-R-modWT (*GTT AAT TTGTGG ATA ACT GTT AAT AAG TTA GGTTTA AAT AGCTAT TTT TAG* GCC AGC CAT TAC GCT CGT) (Figure 3A, step 1). The chimeric primers contained about 51 bp homology (italicized), upstream (CORE6-F) or downstream (CORE6-R-modWT) to the target site on the *Mmc* genome (Additional file 1: Figure S3).

In addition, a second cassette called the knock-in module carrying the 3′ *kanMX* gene component (3′ kanamycin resistance gene sequence (610 to 1357 bp) along with the terminator TEF), a repeat sequence (51 bp homology to the upstream target site of the modified CORE6 knock-out cassette), and the replacement orthologous *Mcc dnaA* gene was constructed in a two-step process (Figure 3A, step 2). Two overlapping PCR amplicons were produced in the first step and assembled in a second step as described below (Figures 2C and 3A). In the first step, the 3′region of the *kanMX* gene component containing a 250 bp overlapping region corresponding to the 5′ sequence of *kanMX* gene component in the CORE6 cassette was generated by PCR using the plasmid pFA6a-kanMX_AJ002680 [27] as the DNA template along with chimeric primers, 3′Kanoverlap + 5′Kan-infusion-F1 (CTG ATG ATG CAT GGT TAC TCA CC) and 3′Kan-repeat-infusion-R1 (ttc *atA TTT GTC TCC AAA AAT ATT ATA GTT AAA CAC TTG TTA AAA ATG TGG AAA ACC AGT ATA GCG ACC AGC ATT C*). The chimeric primer, 3′Kan-repeat-infusion-R1 included a 51 bp repeat sequence (italicized), which is complementary to the upstream sequence from the target site on *Mmc* (*GTT TTC CAC ATT TTT AAC AAG TGT TTA ACT ATA ATA TTT TTG GAG ACA AAT*). Similarly, another PCR fragment containing the orthologous *Mcc dnaA* gene was generated using chimeric primers, repeat-MccdanA-infusion-F2 (*CTA TAC TGG TTT TCC ACA TTT TTA ACA AGT GTT TAA CTA TAA TAT TTT TGG AGA CAA A*ta tga acc taa acg ata ttt taa aag) and Mcc-R1-mod (*GTT AAT TTGTGG ATA ACT GTT AAT AAg tTA GGTTTA AAT AGCTAT TTT TA*T TAT TTT GTT AAA ATT TTATTCTTT AAA ATATCA ACA GTC), and the *Mcc* genomic DNA as template (Figures 2C and 3A). The chimeric primer, repeat-MccdnaA-infusion-F2 included 51 bases complementary to the primer, 3′Kan-repeat-infusion-R1 to create the overlap between the two amplicons. The chimeric primer Mcc-R1-mod included a 50 bp homology to the downstream target site on the *Mmc* genome. In the second step, the linear knock-in module was finally assembled by a PCR-based fusion technique [28] of the two individually synthesized PCR products, the 3′ kanamycin amplicon including the 51 bp repeat, and the replacement *Mcc dnaA* amplicon also carrying the 51 bp repeat sequence (Figures 2C and 3A). All primers were synthesized by Integrated DNA Technologies (Coralville, IA, USA).

#### C. Preparation of mutagenesis cassette for cluster deletion and complementation in the synthetic *Mmc*genome

The CORE6 cassette was PCR-amplified using the plasmid pCORE6 as template, and with chimeric primers D0152/162-F (*AAA ATA AAA ATT CTC TAT AAA ATA TAT TTT GTA AAC TAG AAA GGA AAA GA* T AGG GAT AAC AGG GTA ATA CGG ATT AG) and D0152/162-R (*TTT TTA TTA AAA TAT TTT AAT TAA ATT CAT TAT ATT AAA AGG ATA AAT AA* G GCC AGC CAT TAC GCT CG) (Figure 4A, step 1). In order to introduce the 50 bp repeat sequence (italicized) (*AAA ATA AAA ATT CTC TAT AAA ATA TAT TTT GTA AAC TAG AAA GGA AAA GA)* to the knock-in module, the 3 ′*kanMX* gene component was amplified by two rounds of PCR. In the first round, PCR was performed for 18 cycles using the plasmid pFA6a-kanMX_AJ002680 [27] (Figure 4A, step 2) as the DNA template along with primers, 3′Kan-F (CTG ATG ATG CAT GGT TAC TC) and 3′ Kan-0158-R1 (TCT AGT TTA CAA AAT ATA TTT TAT AGA GAA TTT TTA TTT TCA GTA TAG CGA CCA GCA TT) to generate a 788 bp amplicon. The second round of PCR was conducted for 22 cycles using the 788 bp PCR product as the DNA template along with primers, 3′Kan-F and 3′ Kan-0158-R2 (TTA TTA ATT AAT AAG GAG TAA ATC TTT TCC TTT CTA GTT TAC AAA ATA TAT TTT ATA GA) to generate a 820 bp PCR product where the 50 bp homology (underlined) to the upstream target site was incorporated right after the 3′ *kanMX* gene component. The knock-in gene *Mmc ssrA* gene (679 bp) was amplified by PCR using the synthetic *Mmc* genome (*Mmc* Syn1) [19] as DNA template along with primers, 0158-F ( TAT ATT TTG TAA ACT AGA AAG GAA AAG ATT TAC TCC TTA TTA ATT AAT AAT AAC AA) and 0158-R (TTT TTA TTA AAA TAT TTT AAT TAA ATT CAT TAT ATT AAA AGG ATA AAT AAA CTA ATC AAT CCT AAT AAA TAC TTA G). A final knock-in module (1,527 bp) consisting of the 3′ *kanMX* gene component, the 50 bp repeat sequence, and the *ssrA* gene was assembled by Gibson Assembly method [29]. All primers were synthesized by Integrated DNA Technologies (Coralville, IA, USA).

### Transformation and PCR analysis

Transformation of the modified CORE6 cassette or the knock-in module was performed with lithium acetate as described previously [30]. In all experiments, about 1μg of DNA construct and 25μg of salmon sperm carrier DNA (Sigma, Saint Louis, MO) were used. Transformed yeast were plated on appropriate selection media and incubated at 30°C for 48 hours. Based on the markers present in the DNA cassette and the mycoplasma genome, transformed yeast cells were selected on SD medium minus His (Teknova, CA), SD medium minus His and minus Ura, or YPD containing 0.2 mg/ml geneticin after a period of recovery in YPD (Figure 2).

Yeast colonies growing on selective media were re-streaked and total DNA was isolated for PCR screening [31]. The correct integration of each mutagenesis cassette was verified by PCR screening using diagnostic primers located upstream and downstream of the target sites (Figures 3 and 4 and Additional file 1: Figure S3). All primers were synthesized by Integrated DNA Technologies (Coralville, IA, USA).

### Transplantation

Total DNA, including the intact donor genomic DNA from yeast colonies were isolated using a CHEF Mammalian Genomic DNA Plug Kit as per the manufacturer’s instructions (Bio-Rad, Hercules, CA). DNA isolated from yeast cells carrying the *Mmc* modified genome was transplanted into *Mcc* recipient cells with polyethylene glycol as described previously [21, 31]. The transplanted cells were selected for tetracycline resistance (the *tetM* gene and the β-galactosidase genes (*lacZ*) being present on the *Mmc* chromosome). *Mmc* genomic DNA containing the *Mcc dnaA* gene was isolated from the transplants using the BioRobot M48 workstation (Qiagen, Valencia, CA) as per the manufacturer’s instructions. The isolated *Mmc* genomic DNA from the bacteria transplants was sequenced to confirm the precise, seamless insertion of the *Mcc dnaA* gene (JCVI Sequencing Facility, MD).

## Electronic supplementary material

Additional file 1: Figure S1: Construction of pCORE6 plasmid. The pCORE6 plasmid was constructed from the previously constructed pCORE3 plasmid (unpublished), and the 5′ region of the kanamycin resistance gene (5′ *KanMX* gene component) along with its promoter, PTEF, which was amplified from the previously described pFA6a-KanMX plasmid. The pCORE3 plasmid includes a 14 bp incomplete I-SceI binding site (white bar), a Gal1 promoter, an I-SceI restriction enzyme gene, and yeast KlURA3 prototrophic gene (gray boxes). The plasmid can be selected for HIS3 prototrophy. The pCORE6 also contains a 14 bp incomplete I-SceI site instead of the 18 bp complete sequence for stability reasons, and an additional 4 bp (TAGG) must be added on during PCR for generation of the complete CORE6 knock-out cassette. **Figure S2.** Genes in the two non-essential gene clusters (NEGCs) separated by the Tn5-defined essential gene, *ssrA* in the *Mmc* synthetic genome (*Mmc* Syn1)*.* Genes 0152 – 0157 belong to the first NEGC, while genes 0159–0162 belong to the second NEGC. Gene 0158 is the essential *ssrA* gene that is present between the two NEGCs. **Figure S3.** Diagnostic primers to confirm the correct insertion of the CORE6 knock-out cassette and knock-in cassette by TREC-IN in the *Mmc* genome. Diagnostic primers to assess for the correct junctions and precise insertion of the replaced *Mcc* orthologous *dnaA* gene, and the essential *ssrA* gene in the *Mmc* genome are listed. **Figure S4.** pCORE6 sequence. The CORE6 knock-out cassette (GenBank accession number KP282615) is color-coded as follows: the 14 bp incomplete I-SceI binding site (red), Gal1 promoter (dark green), I-SceI endonuclease (orange), KlURA3 gene along with its promoter and terminator (blue), and the promoter for the translation elongation factor (PTEF) (yellow) followed by the 5′ region of the kanamycin resistance gene (purple). (PDF 251 KB)

## Abbreviations

ARS: Autonomously replicating sequence
DSB: Double strand break
Gal: Galactose promoter
His: Histidine
*KanMX*: Kanamycin resistance gene module
*lacZ*: β-galactosidase genes
Mb: Mega base
*Mcc*: *Mycoplasma capricolum* subspecies *capricolum*
*Mmc*: *Mycoplasma mycoides* subspecies *capri*
*Mmc Syn1*: Synthetic *Mmc* genome
*Mmm*: *Mycoplasma mycoides* subspecies *mycoides*
NEGC: Non-essential gene clusters
NHEJ: Non-homologous end Joining
PCR: Polymerase chain reaction
PTEF: Promoter for the translation elongation factor
SD: Synthetic minimal medium containing Dextrose
TEF: Terminator
*TetM*: Tetracycline resistance
TREC: Tandem repeat coupled with Endonuclease cleavage
TREC-IN: TREC-assisted gene knock-in
Ura: Uracil
YCP: Yeast Centromeric Plasmid
YPD: Rich medium containing glucose
YPG: Rich medium containing Galactose.
