# Supplementary material for: TREC-IN: gene knock-in genetic tool for genomes cloned in yeast
Source: BMC Genomics. 2014 Dec 24;15(1):1180. doi: 10.1186/1471-2164-15-1180 (PMC4407568; doi:10.1186/1471-2164-15-1180)
Supplement: Supplementary file 1 — Additional file 1: Figure S1: Construction of pCORE6 plasmid. The pCORE6 plasmid was constructed from the previously constructed pCORE3 plasmid (unpublished), and the 5′ region of the kanamycin resistance gene (5′ KanMX gene component) along with its promoter, PTEF, which was amplified from the previously described pFA6a-KanMX plasmid. The pCORE3 plasmid includes a 14 bp incomplete I-SceI binding site (white bar), a Gal1 promoter, an I-SceI restriction enzyme gene, and yeast KlURA3 prototrophic gene (gray boxes). The plasmid can be selected for HIS3 prototrophy. The pCORE6 also contains a 14 bp incomplete I-SceI site instead of the 18 bp complete sequence for stability reasons, and an additional 4 bp (TAGG) must be added on during PCR for generation of the complete CORE6 knock-out cassette. Figure S2. Genes in the two non-essential gene clusters (NEGCs) separated by the Tn5-defined essential gene, ssrA in the Mmc synthetic genome (Mmc Syn1). Genes 0152 – 0157 belong to the first NEGC, while genes 0159–0162 belong to the second NEGC. Gene 0158 is the essential ssrA gene that is present between the two NEGCs. Figure S3. Diagnostic primers to confirm the correct insertion of the CORE6 knock-out cassette and knock-in cassette by TREC-IN in the Mmc genome. Diagnostic primers to assess for the correct junctions and precise insertion of the replaced Mcc orthologous dnaA gene, and the essential ssrA gene in the Mmc genome are listed. Figure S4. pCORE6 sequence. The CORE6 knock-out cassette (GenBank accession number KP282615) is color-coded as follows: the 14 bp incomplete I-SceI binding site (red), Gal1 promoter (dark green), I-SceI endonuclease (orange), KlURA3 gene along with its promoter and terminator (blue), and the promoter for the translation elongation factor (PTEF) (yellow) followed by the 5′ region of the kanamycin resistance gene (purple). (PDF 251 KB) [file 12864_2014_6985_MOESM1_ESM.pdf]

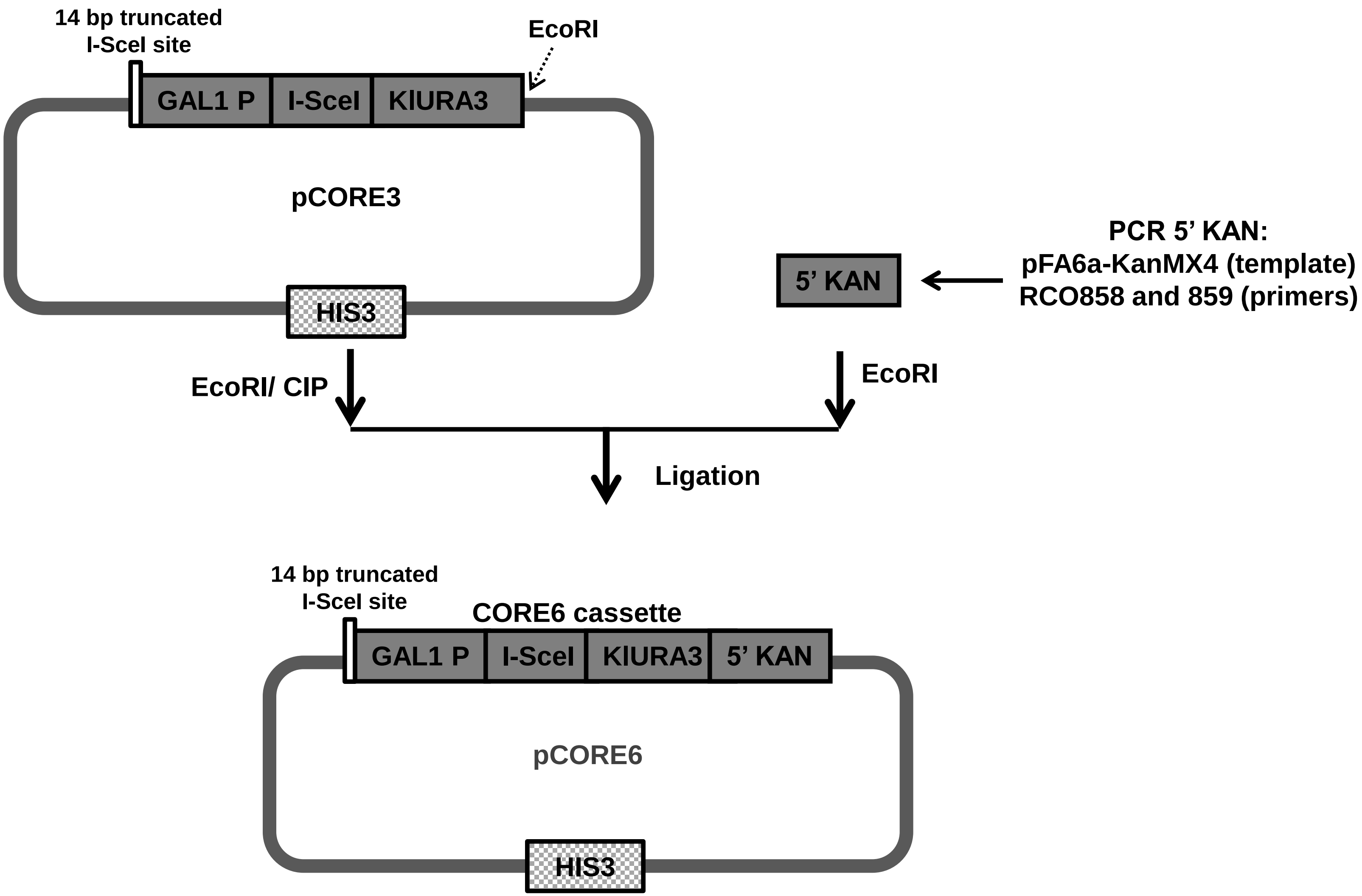
14 bp truncated
I-SceI site
EcoRI
GAL1 P
I-SceI
KlURA3
pCORE3
HIS3
5' KAN
PCR 5' KAN:
pFA6a-KanMX4 (template)
RCO858 and 859 (primers)
EcoRI/ CIP
EcoRI
Ligation
14 bp truncated
I-SceI site
CORE6 cassette
GAL1 P
I-SceI
KlURA3
5' KAN
pCORE6
HIS3

Figure S1

Size (bp)	Gene No.	Annotation
1864	*Mmc* Syn1_0152	PTS system glucose-specific eiic ba component
2269	*Mmc* Syn1_0153	glycosyl hydrolase, family 31
1354	*Mmc* Syn1_0154	leucyl aminopeptidase
601	*Mmc* Syn1_0155	conserved hypothetical protein
664	*Mmc* Syn1_0156	tRNA (guanine-N(7)-)-methyltransferase
1402	*Mmc* Syn1_0157	magnesium transporter
409	*Mmc* Syn1_0158	ssrA
2128	*Mmc* Syn1_0159	putative liporotein
751	*Mmc* Syn1_0160	abortive infection protein AbiGII
595	*Mmc* Syn1_0161	abortive infection protein AbiGI
1205	*Mmc* Syn1_0162	putative lipoprotein

Figure S2

Primer	Primer sequence (5' → 3')	Notes
DG-1F	TAG TTA ATT GTT GAT AAG TTG AT	Diagnostic PCR product (222bp) with primer, DG1-R, diagnostic PCR product (1733bp) with primer, DG2-R, and diagnostic PCR product (338bp) with primer, DG5-R.
DG-1R	GAG TCT TCC TTC GGA GG	Diagnostic PCR product (222bp) with primer, DG1-F.
DG-2F	GCA AAA CAG CAT TCC AGG	Diagnostic PCR product (438bp) with primer, DG2-R.
DG-2R	GGT CAA TTA CTT TAG CTG CTT TTG	Diagnostic PCR product (438bp) with primer, DG2-F, and diagnostic PCR product (1733bp) with primer, DG1-F.
DG-3F	GGA AAG GCT AGA AGT AAA TCA ATT G	Diagnostic PCR product (446bp) with primer, DG2-R.
DG-4R	CAC CAT GAG TGA CGA CTG	Diagnostic PCR product (285bp) with primer, DG2-F.
DG-5R	GTT GGA CGA AAT TGT TTT ATA G	Diagnostic PCR product (615bp) with primer, DG6-F, and diagnostic PCR product (338bp) with primer, DG1-F.
DG-6F	GTT GTA TTG ATG TTG GAC G	Diagnostic PCR product (615bp) with primer, DG5-R.
DG-7F	GTAATCGGTTCAAGTAATGAACAAG	For additional sequencing.
D0152/162-DGF	AAAATAAAAATTCTCTATAAAATATATTTTGTAAACTAGAAAGGAAAAGATAGGGATAACAGGGTAATACGGATTAG	Diagnostic PCR product (230bp) with primer, RCO421, and diagnostic PCR product (1005bp) with primer, D0152/162-DGR.
D0152/162-DGR	TTTTTATTAAAATATTTTAATTAAATTCATTATATTAAAAGGATAAATAAGGCCAGCCATTACGCTCG	Diagnostic PCR product (286bp) with primer, RCO862, and diagnostic PCR product (1005bp) with primer, D0152/162-DGF.
0158-DGF	TATATTTTGTAAACTAGAAAGGAAAAGATTTACTCCTTATTAATTAATAATAACAA	Diagnostic PCR product (283bp) with primer, D0152/162-DGR.
0158-DGR	TTTTTATTAAAATATTTTAATTAAATTCAT	Diagnostic PCR product (239bp) with primer, D0152/162-DGF.
RCO421	CTTCGGAGGGCTGTCACC	Diagnostic PCR product (230bp) with primer, D0152/162-DGF.
RCO862	GTTGCATTCGATTCCTGTTTG	Diagnostic PCR product (286bp) with primer, D0152/162-DGR.

Figure S3

Figure S4 Supplementary sequences: pCORE6

TCGCGCGTTTCGGTGATGACGGTGAAAACCTCTGACACATGCAGCTCCCGGAGACGGTCACAGCTTGTCTGTAAGCGGATGCCGGGAGCAGACAAGCCCGTCAGGGCGCGTCAGCGGGTGTTGGCGGGTGTCGGGG
CTGGCTTAACTATGCGGCATCAGAGCAGATTGTACTGAGAGTGCACCATAATTCCGTTTTAAGAGCTTGGTGAGCGCTAGGAGTCACTGCCAGGTATCGTTTGAACACGGCATTAGTCAGGGAAGTCATAACACAGTCCT
TTCCCGCAATTTTCTTTTTCTATTACTCTTGGCCTCCTCTAGTACACTCTATATTTTTTTATGCCTCGGTAATGATTTTCATTTTTTTTTTTCCACCTAGCGGATGACTCTTTTTTTTTCTTAGCGATTGGCATTATCACATAATGA
ATTATACATTATATAAAGTAATGTGATTTCTTCGAAGAATATACTAAAAAATGAGCAGGCAAGATAAACGAAGGCAAAGATGACAGAGCAGAAAGCCCTAGTAAAGCGTATTACAAATGAAACCAAGATTCAGATTGCGATCT
CTTTAAAGGGTGGTCCCCTAGCGATAGAGCACTCGATCTTCCCAGAAAAAGAGGCAGAAGCAGTAGCAGAACAGGCCACACAATCGCAAGTGATTAACGTCCACACAGGTATAGGGTTTCTGGACCATATGATACATGCT
CTGGCCAAGCATTCCGGCTGGTCGCTAATCGTTGAGTGCATTGGTGACTTACACATAGACGACCATCACACCACTGAAGACTGCGGGATTGCTCTCGGTCAAGCTTTTAAAGAGGCCCTACTGGCGCGTGGAGTAAAAA
GGTTTGGATCAGGATTTGCGCCTTTGGATGAGGCACTTTCCAGAGCGGTGGTAGATCTTTCGAACAGGCCGTACGCAGTTGTCGAACTTGGTTTGCAAAGGGAGAAAGTAGGAGATCTCTCTTGCGAGATGATCCCGCA
TTTTCTTGAAAGCTTTGCAGAGGCTAGCAGAATTACCCTCCACGTTGATTGTCTGCGAGGCAAGAATGATCATCACCGTAGTGAGAGTGCGTTCAAGGCTCTTGCGGTTGCCATAAGAGAAGCCACCTCGCCCAATGGTA
CCAACGATGTTCCCTCCACCAAAGGTGTTCTTATGTAGTGACACCGATTATTTAAAGCTGCAGCATACGATATATATACATGTGTATATATGTATACCTATGAATGTCAGTAAGTATGTATACGAACAGTATGATACTGAAGAT
GACAAGGTAATGCATCATTCTATACGTGTCATTCTGAACGAGGCGCGCTTTCCTTTTTTCTTTTTGCTTTTTCTTTTTTTTTCTCTTGAACTCGACGGATCATATGCGGTGTGAAATACCGCACAGATGCGTAAGGAGAAAAT
ACCGCATCAGGAAATTGTAAACGTTAATATTTTGTTAAAATTCGCGTTAAATTTTTGTTAAATCAGCTCATTTTTTAACCAATAGGCCGAAATCGGCAAAATCCCTTATAAATCAAAAGAATAGACCGAGATAGGGTTGAGTGT
TGTTCCAGTTTGGAACAAGAGTCCACTATTAAAGAACGTGGACTCCAACGTCAAAGGGCGAAAAACCGTCTATCAGGGCGATGGCCCACTACGTGAACCATCACCCTAATCAAGTTTTTTGGGGTCGAGGTGCCGTAAAG
CACTAAATCGGAACCCTAAAGGGAGCCCCCGATTTAGAGCTTGACGGGGAAAGCCGGCGAACGTGGCGAGAAAGGAAGGGAAGAAAGCGAAAGGAGCGGGCGCTAGGGCGCTGGCAAGTGTAGCGGTCACGCTGCG
CGTAACCACCACACCCGCCGCGCTTAATGCGCCGCTACAGGGCGCGTCGCGCCATTCGCCATTCAGGCTGCGCAACTGTTGGGAAGGGCGATCGGTGCGGGCCTCTTCGCTATTACGCCAGCTGGCGAAGGGGGAT
GTGCTGCAAGGCGATTAAGTTGGGTAACGCCAGGGTTTTCCCAGTCACGACGTTGTAAAACGACGGCCAGTGAATTGTAATACGACTCACTATAGGGCGAATTGGAGCTCCACCGCGGTGGCGGCCGCTCTAGAACTAG
TGGATCCCCCGATAACAGGGTAATACGGATTAGAAGCCGCCGAGCGGGTGACAGCCCTCCGAAGGAAGACTCTCCTCCGTGCGTCCTCGTCTTCACCGGTCGCGTTCCTGAAACGCAGATGTGCCTCGCGCCGCACTG
CTCCGAACAATAAAGATTCTACAATACTAGCTTTTATGGTTATGAAGAGGAAAAATTGGCAGTAACCTGGCCCCACAAACCTTCAAATGAACGAATCAAATTAACAACCATAGGATGATAATGCGATTAGTTTTTTAGCCTTAT
TTCTGGGGTAATTAATCAGCGAAGCGATGATTTTTGATCTATTAACAGATATATAAATGCAAAAACTGCATAACCACTTTAACTAATACTTTCAACATTTTCGGTTTGTATTACTTCTTATTCAAATGTAATAAAAGTATCAACAA
AAAATTGTTAATATACCTCTATACTTTAACGTCAAGGAGAAAAAACCCCGGATCCATGCATATGAAAAACATCAAAAAAAACCAGGTAATGAACCTGGGTCCGAACTCTAAACTGCTGAAAGAATACAAATCCCAGCTGATCG
AACTGAACATCGAACAGTTCGAAGCAGGTATCGGTCTGATCCTGGGTGATGCTTACATCCGTTCTCGTGATGAAGGTAAAACCTACTGTATGCAGTTCGAGTGGAAAAACAAAGCATACATGGACCACGTATGTCTGCTGT
ACGATCAGTGGGTACTGTCCCCGCCGCACAAAAAAGAACGTGTTAACCACCTGGGTAACCTGGTAATCACCTGGGGCGCCCAGACTTTCAAACACCAAGCTTTCAACAAACTGGCTAACCTGTTCATCGTTAACAACAAAA
AAACCATCCCGAACAACCTGGTTGAAAACTACCTGACCCCGATGTCTCTGGCATACTGGTTCATGGATGATGGTGGTAAATGGGATTACAACAAAAACTCTACCAACAAATCGATCGTACTGAACACCCAGTCTTTCACTTT
CGAAGAAGTAGAATACCTGGTTAAGGGTCTGCGTAACAAATTCCAACTGAACTGTTACGTAAAAATCAACAAAAACAAACCGATCATCTACATCGATTCTATGTCTTACCTGATCTTCTACAACCTGATCAAACCGTACCTGA
TCCCGCAGATGATGTACAAACTGCCGAACACTATCTCCTCCGAAACTTTCCTGAAATAAGAGTGTTGCACCGTGCCAATGCAGGTGGCTATTAGATTAAATATGTGATTTGTTCTATTAAGTTTCCTGTATAATTAATGGGGA
GCGCTGATTCTCTTTTGGTACGCTTCCCATCCAGCATTTCTGTATCTTTCACCTTCAACCTTAGGATCTCTACCCTTGGCGAAAAGTCCTCTGCCAACAATGATGATATCTGATCCACCACTTACAACTTCGTCGACGGTTCT
GTACTGCTGACCCAATGCATCGCCTTTGTCGTCTAAACCTACACCTGGGGTCATGATTAGCCAATCAAACCCTTCTTCTCTTCCTCCCATATCGTTCTGAGCAATGAACCCAATAACGAAATCTTTATCACTCTTTGCAATAT
CAACGGTACCCTTAGTATATTCACCGTGTGCTAGAGAACCCTTGGAAGACAATTCAGCAAGCATCAATAATCCCCTTGGTTCTTTGGTGACCTCTTGCGCACCTTGTTTCAAGCCAGCAACAATACCAGCACCAGTAACCC
CGTGGGCGTTGGTGATATCAGACCATTCTGCGATACGGTAAACGCCCGATGTATATTGTAATTTGACTGTGTTACCGATATCGGCGAATTTTCTGTCCTCAAATATCAAGAACTTGTATTTCTCTGCCAATGCTTTCAATGGA
ACGACAGTACCCTCATAACTGAAATCATCCAAGATATCAACGTGTGTTTTCAAAAGGCAAATGTATGGACCCAACGTTTCAACAAGTTTCAATAGCTCATCAGTCGAACGAACGTCAAGAGAAGCACACAAATTGGTCTTCT
TTTCATCCATTAAACGTAAAAGTTTCGATGCAACCGGACTTGCATGAGTCTCAGCTCTACTGGTATATGATTTTGTGGACATGGTGCAACTAATTGACGGGAGTGTATTGACGCTGGCGTACTGGCTTTCACAAAATGGCCC
AATCACAACCACATCTTAGATAGTTGAAATGACTTTAGATAACATCAATTGAGATGAGCTTAATCATGTCAAAGCTAAAAGTGTCACCATGAACGACAATTCTTAAGCAAATCACGTGATATAGATCCACGAATAACCACCATT
TGATGCTCGAGGCAAGTAATGTGTGTAAAAAAATGCGTTACCACCATCCAATGCAGACCGATCTTCTACCCAGAATCACATATATTTATGTACCGAGGGCTGCAGGAATTCGACATGGAGGCCCAGAATACCCTCCTTGAC
AGTCTTGACGTGCGCAGCTCAGGGGCATGATGTGACTGTCGCCCGTACATTTAGCCCATACATCCCCATGTATAATCATTTGCATCCATACATTTTGATGGCCGCACGGCGCGAAGCAAAAATTACGGCTCCTCGCTGCA
GACCTGCGAGCAGGGAAACGCTCCCCTCACAGACGCGTTGAATTGTCCCCACGCCGCGCCCCTGTAGAGAAATATAAAAGGTTAGGATTTGCCACTGAGGTTCTTCTTTCATATACTTCCTTTTAAAATCTTGCTAGGATA
CAGTTCTCACATCACATCCGAACATAAACAACCATGGGTAAGGAAAAGACTCACGTTTCGAGGCCGCGATTAAATTCCAACATGGATGCTGATTTATATGGGTATAAATGGGCTCGCGATAATGTCGGGCAATCAGGTGCG
ACAATCTATCGATTGTATGGGAAGCCCGATGCGCCAGAGTTGTTTCTGAAACATGGCAAAGGTAGCGTTGCCAATGATGTTACAGATGAGATGGTCAGACTAAACTGGCTGACGGAATTTATGCCTCTTCCGACCATCAAG
CATTTTATCCGTACTCCTGATGATGCATGGTTACTCACCACTGCGATCCCCGGCAAAACAGCATTCCAGGTATTAGAAGAATATCCTGATTCAGGTGAAAATATTGTTGATGCGCTGGCAGTGTTCCTGCGCCGGTTGCAT
TCGATTCCTGTTTGTAATTGTCCTTTTAACAGCGATCGCGTATTTCGTCTCGCTCAGGCGCAATCACGAATGAATAACGGTTTGGTTGATGCGAGTGATTTTGATGACGAGCGTAATGGCTGGCCGAATTCGATATCAAGCT
TATCGATACCGTCGACCTCGAGGGGGGGCCCGGTACCCAGCTTTTGTTCCCTTTAGTGAGGGTTAATTCCGAGCTTGGCGTAATCATGGTCATAGCTGTTTCCTGTGTGAAATTGTTATCCGCTCACAATTCCACACAACA
TAGGAGCCGGAAGCATAAAGTGTAAAGCCTGGGGTGCCTAATGAGTGAGGTAACTCACATTAATTGCGTTGCGCTCACTGCCCGCTTTCCAGTCGGGAAACCTGTCGTGCCAGCTGCATTAATGAATCGGCCAACGCGC
GGGGAGAGGCGGTTTGCGTATTGGGCGCTCTTCCGCTTCCTCGCTCACTGACTCGCTGCGCTCGGTCGTTCGGCTGCGGCGAGCGGTATCAGCTCACTCAAAGGCGGTAATACGGTTATCCACAGAATCAGGGGATAA
CGCAGGAAAGAACATGTGAGCAAAAGGCCAGCAAAAGGCCAGGAACCGTAAAAAGGCCGCGTTGCTGGCGTTTTTCCATAGGCTCGGCCCCCCTGACGAGCATCACAAAAATCGACGCTCAAGTCAGAGGTGGCGAAA
CCCGACAGGACTATAAAGATACCAGGCGTTCCCCCCTGGAAGCTCCCTCGTGCGCTCTCCTGTTCCGACCCTGCCGCTTACCGGATACCTGTCCGCCTTTCTCCCTTCGGGAAGCGTGGCGCTTTCTCAATGCTCACGC
TGTAGGTATCTCAGTTCGGTGTAGGTCGTTCGCTCCAAGCTGGGCTGTGTGCACGAACCCCCCGTTCAGCCCGACCGCTGCGCCTTATCCGGTAACTATCGTCTTGAGTCCAACCCGGTAAGACACGACTTATCGCCAC
TGGCAGCAGCCACTGGTAACAGGATTAGCAGAGCGAGGTATGTAGGCGGTGCTACAGAGTTCTTGAAGTGGTGGCCTAACTACGGCTACACTAGAAGGACAGTATTTGGTATCTGCGCTCTGCTGAAGCCAGTTACCTT
CGGAAAAAGAGTTGGTAGCTCTTGATCCGGCAAACAAACCACCGCTGGTAGCGGTGGTTTTTTTGTTTGCAAGCAGCAGATTACGCGCAGAAAAAAAGGATCTCAAGAAGATCCTTTGATCTTTTCTACGGGGTCTGACG
CTCAGTGGAACGAAAACTCACGTTAAGGGATTTTGGTCATGAGATTATCAAAAAGGATCTTCACCTAGATCCTTTTAAATTAAAAATGAAGTTTTAAATCAATCTAAAGTATATATGAGTAAACTTGGTCTGACAGTTACCAAT
GCTTAATCAGTGAGGCACCTATCTCAGCGATCTGTCTATTTCGTTCATCCATAGTTGCCTGACTGCCCGTCGTGTAGATAACTACGATACGGGAGGGCTTACCATCTGGCCCCAGTGCTGCAATGATACCGCGAGACCCA
CGCTCACCGGCTCCAGATTTATCAGCAATAAACCAGCCAGCCGGAAGGGCCGAGCGCAGAAGTGGTCCTGCAACTTTATCCGCCTCCATCCAGTCTATTAATTGTTGCCGGGAAGCTAGAGTAAGTAGTTCGCCAGTTAA
TAGTTTGCGCAACGTTGTTGCCATTGCTACAGGCATCGTGGTGTCACGCTCGTCGTTTGGTATGGCTTCATTCAGCTCCGGTTCCCAACGATCAAGGCGAGTTACATGATCCCCCATGTTGTGAAAAAAAGCGGTTAGCTC
CTTCGGTCCTCCGATCGTTGTCAGAAGTAAGTTGGCCGCAGTGTTATCACTCATGGTTATGGCAGCACTGCATAATTCTCTTACTGTCATGCCATCCGTAAGATGCTTTTCTGTGACTGGTGAGTACTCAACCAAGTCATTC
TGAGAATAGTGTATGCGGCGACCGAGTTGCTCTTGCCCGGCGTCAATACGGGATAATACCGCGCCACATAGCAGAACTTTAAAAGTGCTCATCATTGGAAAACGTTCTTCGGGGCGAAAACTCTCAAGGATCTTACCGCT
GTTGAGATCCAGTTCGATGTAACCCACTCGTGCACCCAACTGATCTTCAGCATCTTTTACTTTCACCAGCGTTTCTGGGTGAGCAAAAACAGGAAGGCAAAATGCCGCAAAAAAGGGAATAAGGGCGACACGGAAATGTT
GAATACTCATACTCTTCCTTTTTCAATATTATTGAAGCATTTATCAGGGTTATTGTCTCATGAGCGGATACATATTTGAATGTATTTAGAAAAATAAACAAATAGGGGTTCCGCGCACATTTCCCCGAAAAGTGCCACCTGGGT
CCTTTTCATCACGTGCTATAAAAATAATTATAATTTAAATTTTTTAATATAAATATATAAATTAAAAATAGAAAGTAAAAAAAGAAATTAAAGAAAAAATAGTTTTTGTTTTCCGAAGATGTAAAAGACTCTAGGGGGATCGCCAA
CAAATACTACCTTTTATCTTGCTCTTCCTGCTCTCAGGTATTAATGCCGAATTGTTTCATCTTGTCTGTGTAGAAGACCACACACGAAAATCCTGTGATTTTACATTTTACTTATCGTTAATCGAATGTATATCTATTTAATCTG
CTTTTCTTGTCTAATAAATATATATGTAAAGTACGCTTTTTGTTGAAATTTTTTAAACCTTTGTTTATTTTTTTTTCTTCATTCCGTAACTCTTCTACCTTCTTTATTTACTTTCTAAAATCCAAATACAAAACATAAAAATAAATAAA
CACAGAGTAAATTCCCAAATTATTCCATCATTAAAAGATACGAGGCGCGTGTAAGTTACAGGCAAGCGATCCGTCCTAAGAAACCATTATTATCATGACATTAACCTATAAAAATAGGCGTATCACGAGGCCCTTTCGTC
